# A Multi-Point Sensor Based on Optical Fiber for the Measurement of Electrolyte Density in Lead-Acid Batteries

**DOI:** 10.3390/s100402587

**Published:** 2010-03-25

**Authors:** Ana M. Cao-Paz, Jorge Marcos-Acevedo, Alfredo del Río-Vázquez, Carlos Martínez-Peñalver, Alfonso Lago-Ferreiro, Andrés A. Nogueiras-Meléndez, Jesús Doval-Gandoy

**Affiliations:** Department of Electronic Technology, University of Vigo, 36310 Vigo, Spain; E-Mails: ario@uvigo.es (A.D.R.V.); penalver@uvigo.es (C.M.P.); alago@uvigo.es (A.L.F.); aaugusto@uvigo.es (A.A.N.M.); jdoval@uvigo.es (J.D.G.)

**Keywords:** plastic optical fiber, bending losses, electrolyte density, liquid level, lead acid batteries, state of charge, battery maintenance

## Abstract

This article describes a multi-point optical fiber-based sensor for the measurement of electrolyte density in lead-acid batteries. It is known that the battery charging process creates stratification, due to the different densities of sulphuric acid and water. In order to study this process, density measurements should be obtained at different depths. The sensor we describe in this paper, unlike traditional sensors, consists of several measurement points, allowing density measurements at different depths inside the battery. The obtained set of measurements helps in determining the charge (SoC) and state of health (SoH) of the battery.

## 1. Introduction

Many electrical systems need batteries to be operational; some because of the impossibility of being permanently connected to a power supply and others in order to improve their mobility. Among electrochemical accumulators, lead-acid batteries are one of the most widely used types [1]. They are found in many fields such as the automotive industry—where lead-acid batteries have been used since the very beginnings—as energy storage for solar panels, in various telecommunication applications, for remote systems, in submarines, and many others.

Most applications require a reliable measurement of battery state in order to improve the performance of the equipment by planning the available operating time or detecting faults as soon as possible. A malfunctioning battery may cause large economic losses or in the case of more sensitive equipment, even the loss of human lives.

The main parameters in the assessment of a battery are current, voltage, temperature, state of charge (SoC) and state of health (SoH). The state of charge is one of the most significant parameters of a battery. It provides key information that is useful for the improvement of battery operation, performance, reliability and life span. Knowing the state of charge it is possible to avoid an overcharging that would lead to a decrease in working life or even to a battery malfunction [2,3].

The measurement of electrolyte density provides an accuracy value of battery SoC. The lead-acid battery uses lead dioxide (PbO_2_) as the active material in the positive electrode, and metallic lead (Pb) of a very porous structure, as the active material in the negative electrode. The electrolyte is formed by sulphuric acid (H_2_SO_4_) diluted in water (H_2_O), with concentrations between 8% and 40%, depending on the state of charge and the type of battery. The electrochemical reactions which occur during the process of charging and discharging are described in Equations (1) to (6):

During the charge:

(1) $$\text{Anode}\;(+):{\text{PbSO}}_{4}+2H_{2}O\to {\text{PbO}}_{2}+H_{2}{\text{SO}}_{4}+2H^{+}+2e^{-}$$

(2) $$\text{Cathode}\;(-):{\text{PbSO}}_{4}+2e^{-}\to {\text{SO}}_{4}^{2-}+\text{Pb}$$

(3) $$\text{Global}\;\text{reaction}:2\;{\text{PbSO}}_{4}+{2H}_{2}O\to \text{Pb}+{\text{PbO}}_{2}+2H_{2}{\text{SO}}_{4}$$

During the discharge:

(4) $$\text{Anode}\;(+):\text{Pb}+{\text{SO}}_{4}^{2-}\to {\text{PbSO}}_{4}+2e^{-}$$

(5) $$\text{Cathode}\;(-):{\text{PbO}}_{2}+H_{2}{\text{SO}}_{4}+2H^{+}+2e^{-}\to {\text{PbSO}}_{4}+2H_{2}O$$

(6) $$\text{Global}\;\text{reaction}:\text{Pb}+{\text{PbO}}_{2}+2H_{2}{\text{SO}}_{4}\to 2\;\;{\text{PbSO}}_{4}+2H_{2}O$$

According to Equations (4) to (6), during the discharging process both electrodes transform the active material into lead sulphate (PbSO_4_), with the consequent consumption of H_2_SO_4_ and the release of water to the electrolyte. As a result the electrolyte density decreases. In the charging process, Equations (1) to (3), the opposite reaction occurs since H_2_SO_4_ is released and water is consumed, thereby causing an increase in the electrolyte density.

Since the refractive index depends on electrolyte density [1], the former can be used to assess the latter. In addition, the evolution density *versus* time curve is a good indicator of battery SoH.

Density sensors are based on different principles and may be classified as hydrostatic, electrochemical or electrical, optical [4–7] and, a final group that includes sensor based on various properties such as ion exchange membrane [8] or electrolyte conductivity [9]. These methods have been studied by several researchers and they all allow measurement of density, but not all of them are suitable for our application. Within the category of optical sensors, the optical fiber sensors have proved to be suitable for many applications and since the 1970s multiple configurations have been developed for applications in physical, chemical, environmental, mechanical measurements, *etc.*, [10]. The properties of plastic optical fiber have enabled the development of more robust, economical and versatile sensors [11–15].

Several optical fiber sensors have been tested in measurements of the electrolyte density in batteries. The devices proposed in [6] and [12] allow a density measurement of the electrolyte by means of optical fiber refractometers, but, due their large size the desirable density measurements at different heights are not possible. In the same way, traditional sensors only measure density in the upper part of the battery. This measurement is not representative, due to the stratification process in the electrolyte which produces a density gradient during the charge. In this process, the sulphuric acid from the plates is deposited in the bottom of the battery, because its density is higher than of the electrolyte [1]. This situation lasts until, at the end of the charge (70–80%), bubbling is produced and density homogenizes, leading to a non-linear density variation curve with respect to the SoC [1]. This prevents the density of the battery from being estimated in real time during the charging process. During the discharge, the relation between the percentage of charge and the density of the electrolyte is linear. Some researchers have developed alternative methods to obtain a uniform density in the whole cell by means of the circulation of the electrolyte [16].

This article presents a sensor based on plastic optical fiber which can be easily introduced between the plates of the battery. Its main advantage is that the measurement of density is carried out at different levels inside the container of the battery, which allows monitoring of the density in real time and in the whole cell during charging and/or discharging process. Since the measurements are taken by introducing the fibers inside the cells, there is no need to use auxiliary automated systems that make the electrolyte circulate or periodically extract a sample of electrolyte. These systems, besides increasing the cost of measurement, take up a lot of space, are a possible source of noise and, in general, complicate the monitoring of the parameters.

This article is divided into four parts. In the first part the operating principles of the optical fiber sensor is presented. The second part describes the development phases of the one-point sensor, the results obtained with this configuration, and its validity for comparison with other commercial sensors. In the third part the multi-point sensor is dealt with in detail. The advantages over traditional sensors are also shown, including the use of one sensor as an electrolyte level meter. Finally, in the fourth part, a 7,000 hour accelerated testing of the plastic optical fibers is presented in order to show that the developed fiber sensor is suitable for the proposed application and resists the corrosion of the medium (electrolyte).

## 2. Operating Principles

The sensor consists in a U-shaped plastic optical fiber which is jacketless around the bend, so as to allow the fiber cladding to be in direct contact with the liquid whose density is to be measured. The propagation of light through optical fiber has been studied by several authors [17]. Snell’s law for a refracted ray is fulfilled according to Equation 7:

(7) $$n_{co}\;\text{cos}\;\theta_{z}=n_{cl}\;\text{cos}\;\theta_{t}$$

where *θ_z_* is the incident angle in the limit between the two media with a different index of refraction and *θ_t_* is the angle of the refracted ray. The total reflexion is given if 0 ≤ *θ_z_* ≤ *θ_c_* and the ray is partially refracted when *θ_c_* < *θ_z_* ≤ π/2. *θ_c_* is the complement of the critical angle, defined by Equation 8; *n_co_* is the index of refraction of the core and n_cl_ is the index of refraction of the cladding:

(8) $$\theta_{c}={\text{cos}}^{-1}\left[\frac{n_{cl}}{n_{co}}\right]={\text{sin}}^{-1}{\left[1-\frac{n_{cl}{}^{2}}{n_{co}{}^{2}}\right]}^{1/2}$$

The propagation of the power of light through the route of the refracted ray has losses, due to the power transmitted to the jacket. These losses are represented by the coefficient of transmission of the power T, or coefficient of losses, Equation 9:

(9) $$T=1-\left(\frac{\mathrm{power}\;\mathrm{in}\;\mathrm{the}\;\mathrm{reflected}\;\mathrm{ray}}{\mathrm{power}\;\mathrm{in}\;\mathrm{the}\;\mathrm{incident}\;\mathrm{ray}}\right)$$

**Figure 1.**
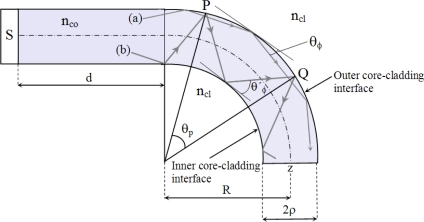
Guided rays in a curved zone of a planar waveguide.

In a planar waveguide, with a step index and with a curved section [18], the routes of the rays trace a straight line between the reflexions of the external interface as shown in the route (b) of Figure 1; there are other possible routes (a) whose reflexions only appear in the external interface (whispering gallery rays).

The attenuation of power in a curved fiber has been widely studied [18–23]. The classic coefficient of transmission of Fresnel has been used for the refracted rays:

(10) $$T_{r}=\frac{4\;\text{sin}\;\theta_{z}{\left({\text{sin}}^{2}\;\theta_{z}-{\text{sin}}^{2}\;\theta_{c}\right)}^{½}}{{\left[\text{sin}\;\theta_{z}+{\left({\text{sin}}^{2}\theta_{z}-{\text{sin}}^{2}\;\theta_{c}\right)}^{½}\right]}^{2}}$$

where θc depends on the indices of refraction of the core and of the cladding (8). A coefficient of different transmission (*T_t_*) should be used for the tunneling rays [17]. However, for the typical values of the parameters (θ_z_ y θ_c_), *T_t_* ≈10^−8^; therefore, it may be considered that there are no losses for tunneling except for a few angles with a value close to the critical angle [24].

**Figure 2.**
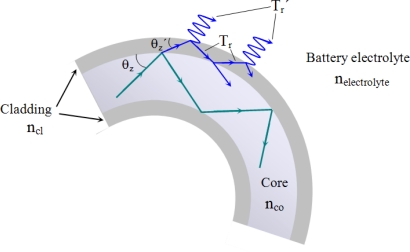
Loss of power taking into account the thickness of the cladding.

The rays which are refracted in the external interface, core-cladding, may be guided by successive reflexions between the inner core-cladding interface and the cladding-medium external interface due to the differences between the indices of refraction n_cl_-n_co_ y n_cl_-n_medium_ (Figure 2). In the points in which the rays reach in the external interface cladding-medium, there is a loss of power because of the refraction to the external medium, which, in our case, is the electrolyte of the battery. In order to evaluate the losses at these points, the Fresnel coefficient of Equation (11) is used, but with the values of the angle of incidence *θ_z_′* and of the complementary values of the critical angle *θc′* (12). Then, according (11) and (12), the losses of the power in the bend of the fiber depend on the index of refraction of the electrolyte of the battery:

(11) $$T_{r}{}^{\prime }=\frac{4\;\text{sin}\;\theta_{z}{}^{\prime }{\left({\text{sin}}^{2}\;\theta_{z}{}^{\prime }-{\text{sin}}^{2}\;{\theta^{\prime }}_{c}\right)}^{½}}{{[\text{sin}\;{\theta^{\prime }}_{z}+{\left({\text{sin}}^{2}\;\theta_{z}{}^{\prime }-{\text{sin}}^{2}\;{\theta^{\prime }}_{c}\right)}^{½}]}^{2}}$$

(12) $$\theta_{c}{}^{\prime }={\text{cos}}^{-1}\left(\frac{n_{\mathrm{electrolyte}}}{n_{\mathrm{cl}}}\right)$$

## 3. Optical Fiber Point Sensor

### 3.1. Sensor configuration

The characteristics of the plastic optical fiber (POF—Polymer optical fiber) provide many advantages that allow the manufacture of more robust, economical and versatile sensors [11,25], including low attenuation at the visible range of spectrum, wide light acceptance angle, excellent mechanical and environmental properties that produce durability and reliability. The fiber can be bent without breaking, and it is chemically neutral. The alignment between light source and fiber is not critical. All these properties lead us to use plastic optical fibers.

The presented sensor includes a step-index 1-milimeter plastic optical fiber (SH4001). This fiber has a concentric double-layer structure with a high-purity polymethyl methacrylate (PMMA) core and a thin layer of transparent fluorine polymer cladding. The cladding has a lower refractive index than that of the core (*n_co_* = 1.49, numerical aperture = 0.5). The core diameter is 920–1040 μm and the cladding diameter is 940–1,060 μm. The fiber jacket material is polyethylene and its outer diameter is 2.2 mm.

The first step in building the sensor is to remove the jacket from the middle section of a fiber (called sensitive fiber) along 20–30 mm, using a stripper and cutting tools (special designed for POF). In this section, as soon as the jacket is removed, the fiber cladding is uncovered. Since this procedure is manual and cladding thickness is very small, some damage is likely to occur in the cladding or even in the core. Therefore, this procedure should be done carefully, and the section examined using an electronic magnifier in order to detect any damage in the cladding or in the core.

Later, the fiber is bent in a U-shape, with the cladding exposed section (sensitive zone) just in the middle of the U. The optical emitter and receiver are connected to the respective ends of the fiber using appropriate connectors. The light coming from the emitter is sent through the fiber core by total reflexion to the sensitive zone. In this zone, the light is partially refracted to the cladding and, since the fiber jacket was removed, partially refracted to the external medium, according to the principle of operation shown in Section 2. The remaining light is transmitted to the other end of the fiber, where the photo-receiver is placed.

**Figure 3.**
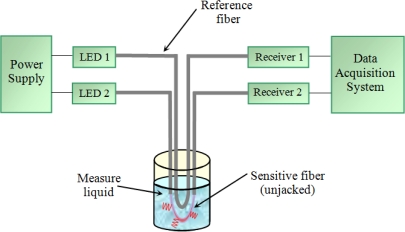
Diagram of the optical fiber sensor.

A second fiber is added to the sensor as a reference, in order to compensate for some possible variations in the supply voltage or other elements. This fiber is bent in a U-shape too, but its protective jacket is preserved. The reference fiber and the sensitive fiber share the same path and the same common variations such as temperature, movements, light fluctuations, *etc*. However, only the sensitive fiber is affected by refraction, since the reference fiber cladding is not in contact with the electrolyte. Figure 3 shows a diagram of the optical fiber sensor.

As a part of the sensor development process, the U radius is modified in order to optimize its sensitivity, which is one of the critical parameters of the sensor. The fiber technical data show a minimum radius without losses of 25 mm. In this application, however, the aim is precisely to obtain a loss in the curvature. Therefore several experiments were carried out in order to obtain the optimal radius for this application.

Six fibers with radii between 1 and 10 mm were immersed in electrolyte solutions with different concentration of H_2_SO_4_ in the range of the battery state of charge. The experimental procedure was: firstly, fibers with a large radius (10 mm) were immersed in solutions with different density. When all the solutions were tested, the radii were diminished and the fibers were tested again with all the solutions. This procedure was repeated for each radius until the minimal value (1 mm). The output voltage of the receivers decreases when the solution density increases; all the radii tested exhibit this behavior. However, the voltage difference between the most dense and the least dense solutions depends on the radius length. The optimal radius is considered to be the one that provides the greater output voltage difference between the most and least dense medium, that is, the most sensitive radius within the expected range of electrolyte density.

**Figure 4.**
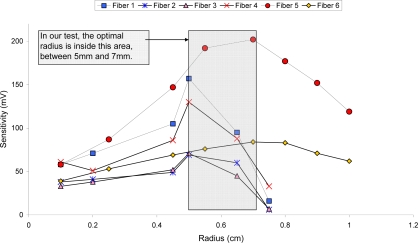
Determination of the radius length that provides the highest sensitivity.

**Figure 5.**
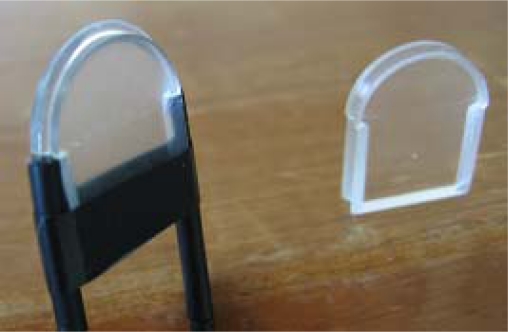
Optical fiber placed on a 6-milimeter-radius mould.

Figure 4 shows the response for the different radius values tested, Y-axis represents the sensitivity (difference in output voltage) within the range of electrolyte density. Due sensitivity is greatest within the 5 to 7 mm range [26], moulds with a radius length of 6 mm were made in order to simplify the design process, as shown in Figure 5.

The optimal radius sensor is suitable for sensors which are placed at the top of the battery. For lower positions between the plates, the design of the battery should be adapted properly. When the fiber sensor with the optimal radius is to be introduced inside an operational standard battery, as it was the case in the present project, the process is very difficult or even impossible to implement depending on the type of battery. Therefore, if the sensor has to be placed by hand, a radius length below the optimum must be used because the distance between the battery plates is too small or even non-existent in some types of batteries

Using traction batteries, the placement of fiber (with a minimum radius) between the plates is possible due to the longer distance between the plates. The introduction of fibers in a battery is carried out manually by means of a transparent container; allowing visual inspection of fiber location. A radius length of 1.5 mm was selected as a trade-off between sensitivity and handiness.

The sensitivity of the 1.5-milimeter-radius sensor is low, but high enough to be useful. Some experiments have confirmed its validity using the mutipoint sensor, which is discussed later.

### 3.2. Electronic system

Once the optical fiber is defined, the optoelectronic devices are selected based on the fiber characteristics. Since plastic optical fibers exhibit light attenuation windows at certain wavelengths [25], especially in the infrared range, a 650-nanometer LED was selected.

Device packages have been selected in order to simplify the coupling between device and fiber. The HFBR 1527 transmitter and the HFBR 2526 receiver were selected following this aspect and the reliability they have shown in previous projects.

**Figure 6.**
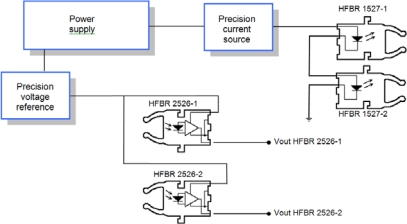
Electronic circuit block diagram with two emitter-receiver pairs, one for the sensitive fiber and another for the reference fiber.

The HFBR 2526 includes an additional circuit and provides an analog output voltage. This simplifies the design of the external circuit associated with the receiver. Figure 6 shows a block diagram of the sensor electronic circuit including two emitters and two receivers (sensitive and reference fibers). The only requirement for the receivers is the availability of a very stable supply voltage.

In order to stabilize the light emitted by LEDs, their bias current is controlled by an accurate current source. Since both LEDs are connected in series, they share the same bias current. This way, light fluctuations are similar in both fibers allowing the use of some compensation system.

The system has two major drawbacks. Firstly, the weakness in the connections between the optical devices and the fiber creates instability in the receiver output voltage, with any slight movement. This problem has been first addressed by immobilizing the fibers and the coupling areas as much as possible. In addition, new tests are also underway using devices with different coupling systems. Secondly, the selected components (especially the receivers) have a strong dependence on temperature which corrupts the measurements. As a consequence, some compensation and suppression methods have been developed. These methods are shown in the next section.

The whole electronic system also includes two PT100-type temperature sensors for both, the ambient temperature and the battery temperature measurements.

**Figure 7.**
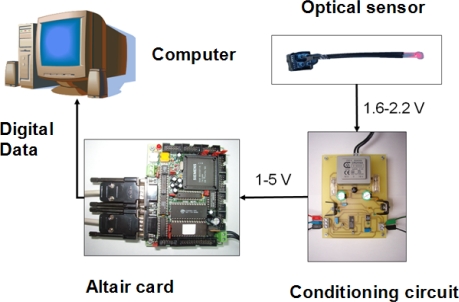
Diagram of the Visualizing system of optoelectronic sensors.

In order to improve the sensor, a conditioning and visualization system has been developed, which receives the signal from the sensor and adapts its levels to standard values. The adapted signal is sent to an Altair card based on the Siemens SAB80C537 microcontroller, which transforms the signal received into data, and sends them to the computer via a serial port. The computer shows the data using suitable software, specifically designed for this application. In Figure 7, a diagram of the electronic system is shown.

### 3.3. Temperature compensation

**Figure 8.**
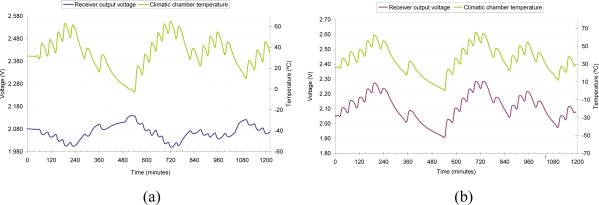
(a) Receiver output voltage in emitter thermal test. (b) Receiver output voltage in receiver thermal test.

The sensor is considerably affected by a change in temperature. This is due mainly to the effect that temperature has on optical emitters and receivers. As a consequence, several thermal tests have been carried out separately on the emitter and the receiver, as well as on the overall receiver transmitter circuit [27]. The first two tests are depicted in Figure 8, showing a negative thermal drift in the emitter (8a), whereas the receiver shows a positive thermal drift (8b). The receiver dependency on temperature is greater than of the emitter.

When the emitter temperature changes by using a climatic chamber, the receiver is maintained at a constant temperature in another climatic chamber and they are connected by the fiber. Similarly, the emitter is at a constant temperature when the receiver temperature changes. In both tests the receiver output is obtained.

#### 3.3.1. Temperature compensation by means of Data Processing

From the tests shown in the previous section, some correction coefficients have been extracted. Applying these coefficients to the output voltage of the receiver, thermal drift can be almost completely corrected. Unfortunately the parameter values are specific to a specific sensor. Therefore, a test has to be carried out for every single sensor in order to obtain its characterization. In addition, any change in the sensor structure—the radius length of the fiber U-shape, the fiber connections or other components—makes necessary a new test.

The reference fiber provides an alternative method for temperature compensation. This method is easy to implement and its results are better than those of the correction by thermal coefficients. Subtracting the output voltage of the reference fiber from the output voltage of the sensitive fiber, a good temperature correction is obtained.

**Figure 9.**
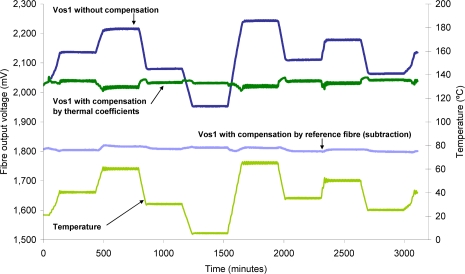
Compensation by data processing, comparison between the compensation by the reference fiber and the thermal coefficients method.

Figure 9 shows both methods for comparison. A sensitive fiber is tested in air within a temperature range between 5 and 65 °C (Tc). Vos1 represents the fiber output voltage without compensation. These data are processed using both methods: the compensation with the reference fiber and the correction with the thermal coefficients.

The method of compensation by the reference fiber, in addition to good thermal correction, provides the correction of other common mode factors such as fluctuations in light source, movements, *etc*. In order to obtain a successful correction, the reference fiber should operate under the same conditions as the sensitive fiber, including a common path, common fluctuations, the same injected light level, (diodes in series), the same temperature, *etc*. If it is not possible for both fibers to share the same conditions, or if the reference fiber is removed, the thermal coefficients method is still a good choice.

#### 3.3.2. Temperature compensation by hardware

**Figure 10.**
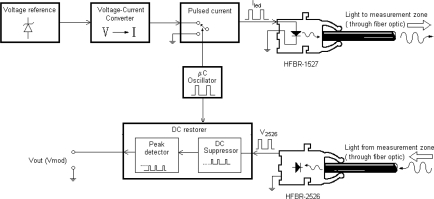
Block Diagram of the system for temperature compensation by means of hardware

In order to eliminate temperature dependence, the compensation by hardware system depicted in Figure 10 was carried out.

One of the main advantages of the circuit is that it is valid for any optical fiber sensor and a previous calibration is not necessary. In addition; the influence of environmental light is cancelled due to the DC suppressor. The main drawback of this circuit is that it compensates the thermal drift of the receiver only. It does not take into account the thermal drift produced by the emitter.

**Figure 11.**
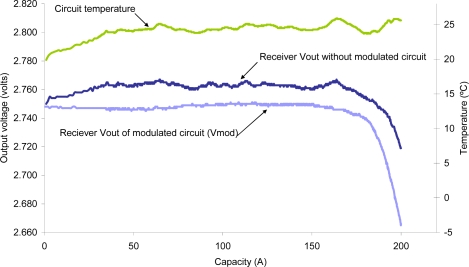
Compensation of temperature in the receptor by means of hardware.

Figure 11 shows a graph obtained using the compensation by hardware method. The original fiber measurement (receiver output voltage) is affected by temperature, but this effect is cancelled using the modulated circuit described below.

In summary, temperature compensation methods that are applied to the sensor give a good correction. The correction by thermal coefficients has one major drawback, it is necessary to perform a thermal characterization of each individual fiber, and if conditions change, it is necessary to calculate the new coefficients. Moreover, the compensation with the reference fiber is a very simple method to implement and the results are good, if both fibers are subject to the same conditions (except the measurement itself). Finally, the hardware compensation also corrects the influence of ambient light but does not include compensation in the transmitter. In our case, the method most widely used and most successful is the compensation with the reference fiber.

### 3.4. Results

**Figure 12.**
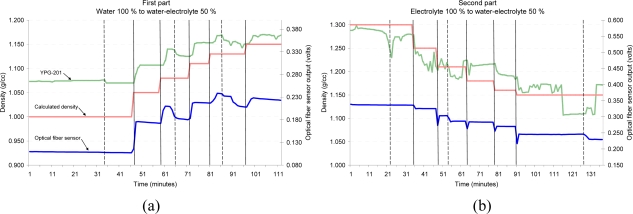
Behavior of the optical fiber sensor in solutions of electrolyte and water. (a) Water 100% to water-electrolyte 50%. (b) Electrolyte 100% to water –electrolyte 50%.

Tests were carried out with solutions of electrolyte and water. The test consists of two parts. The first part (Figure 12a) begins using a 100% water solution. Then, electrolyte is added at regular intervals until a concentration of 50% water-electrolyte volume is obtained. In the second part (Figure 12b) the test begins using a 100% electrolyte solution, and then adding water until a fifty-fifty concentration is reached. During the experiment, bubbles are produced artificially in order to simulate the bubbling process which occurs in lead-acid batteries during the charging process (especially in fast charge and at the end of the charge), so that the behavior of the sensor can be studied under these conditions. In order to check the validity of the measurements, a YUASA density meter, model YPG-201 is used as a density reference.

The vertical dashed lines represent the instant when the bubbles are produced, and the solid lines indicate the instant when electrolyte or water is added. The real density of the electrolyte is also represented in Figure 12, and it was calculated taking into account the existing proportions of electrolyte and water at any time and the theoretical density for both. It is observed that when adding the electrolyte to water, the fiber sensor output firstly decreases its value, and then increases. This behavior is due to the deposition of the electrolyte in the bottom of the recipient (denser), which makes the sensor to detect lower density. The YPG-201 optical density meter shows a similar behavior, as both sensors are placed at the same height in the recipient. The curves of real density do not take into account the heterogeneity of the mixture.

**Figure 13.**
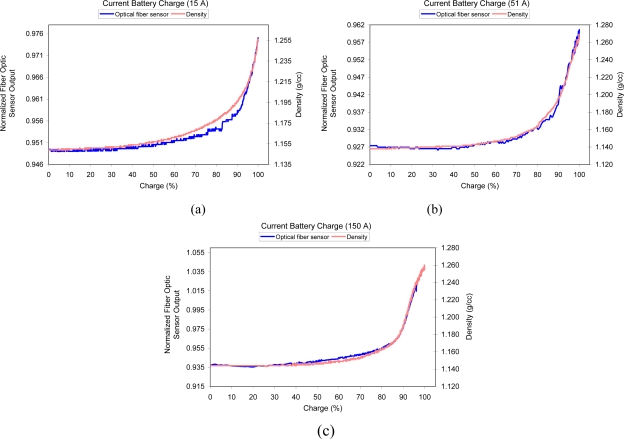
Measurement of the density of the electrolyte during the battery charging process taken by the optical fiber sensor (normalized values) and using the YUASA sensor YPG-201 (gr/cc). (a) Charge 15 A, (b) Charge 51 A. (c) Charge 150 A.

In order to verify the correct operation of the optical fiber in real measurements in batteries, tests were carried out during the processes of charging and discharging using a testing bench which includes an automated system for the charge and discharge of batteries with different charging regimes [28,29]. Figure 13 shows the real time results for a slow charge (15 A), medium charge (51 A) and fast charge (150 A). These results are based on the measurements obtained from the upper part of the battery.

**Figure 14.**
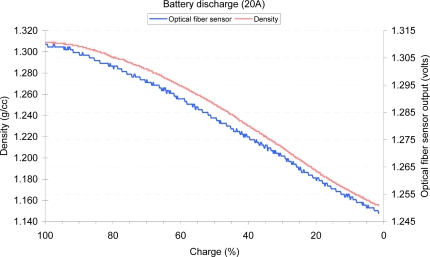
Measurements of the density of the electrolyte during the battery discharging process obtained from the optical fiber sensor (volts) and from the YUASA YPG-201 (gr/cc) sensor.

Figure 13 shows the effects of the stratification of the electrolyte during the battery charging process. The stratification makes the SoC to not vary linearly with the density of the electrolyte in the upper part of the battery. A discharge experiment is shown in Figure 14. Both experiments show a good correlation between the optical fiber sensor and the YPG-201 density meter.

The effect of temperature on the devices is compensated by the reference fiber, although in some experiments a redundant sensor with hardware compensation is also tested for comparison. The thermal drift of the density is also corrected, which, in the case of the electrolyte is −0.0007 kg °C/cc. In these measurements the YUASA density meter was used again as a reference in order to validate the results.

## 4. Multi-Point Sensor

### 4.1. Measurement of density at several points

In order to carry out measurements at several points, a sensor was made using five optical fibers. Four of them are sensing fibers, while the other is a reference fiber. The sensing fibers are used to take measurements of density of the electrolyte at different heights inside the battery. This multi-point sensor shows several benefits if compared to commercial sensors which only measure the density in the upper part of the battery. The most significant benefit is that, due to the multi-point sensor, it is possible to determine the SoC of the battery in real time, whereas the sensors which only measure the density in the upper part of the battery cannot.

Due to the stratification of the density of the electrolyte during the battery charging process, the electrolyte density becomes homogeneous only after some time. Therefore the SoC of the battery cannot be accurately stated in real time. However, the multipoint sensor makes it possible to monitor the density at several heights so that the stratification of the electrolyte can be detected in real time.

**Figure 15.**
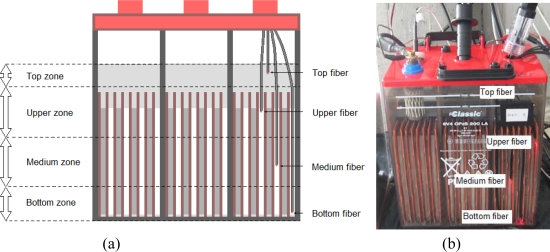
Multi-point sensor inside the battery. (a) Identification of different zones. (b) Photograph of the sensor located inside the cell.

As mentioned before, the sensor consists of five fibers, four of which are used for measuring the density. In order to place the fibers, the battery is divided into four zones: top zone, upper zone, middle zone and bottom zone; a sensor is placed in each one of these four zones, as shown in Figure 15a. Figure 15b shows the fibers introduced inside a real battery with a transparent container. As noted above, the optimal radius can not be used in this configuration because it is impossible to manually position the fibers inside the battery. As a result, the radius length of 1.5 mm is used, and the sensitive is lower. Fortunately, this does not affect measurements significantly.

For reference density measurement, calibration is performed in situ. Periodically, samples of electrolyte were taken from the same points where each fiber carries out the measurement. With the precision density meter DMA-35 the density was measured and, immediately, the samples went back to the battery in order to not cause a decrease in the fluid level.

**Figure 16.**
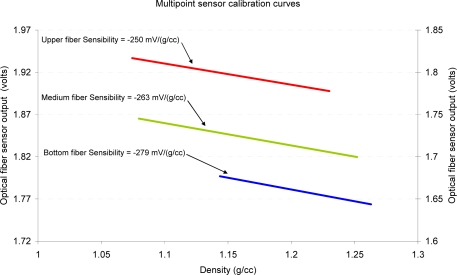
Calibration plot of the multipoint sensor in the bottom, medium and upper zones of the battery.

The calibration plot obtained for the upper, medium and bottom fibers is shown in Figure 16. It can be seen that the bottom fiber does not exhibit values lower than 1.14 g/cc, it is because the density in this area has not dropped below that value in any of the calibration tests performed. We observed hysteresis of 10% over the full range of the sensor output. This value corresponds with the fiber placed in the upper zone of the battery and it is given in the ascending trend of the input variable from 10% to 40% of load.

**Figure 17.**
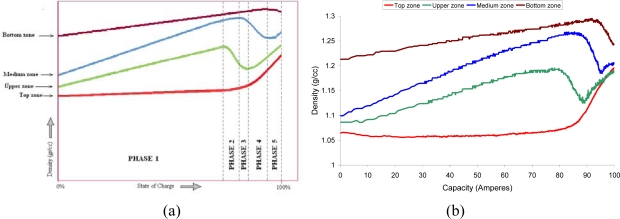
(a) The typical trend of the density in the four zones into which the battery is divided. (b) Real time density of the battery measured with the multi-point sensor during the real charging process.

The multi-point sensor was tested in a multitude of charges and discharges, it was observed that the evolution of the density was repetitive and unique in terms of the height at which the measurement was performed. Based on the trends observed in the fibers during the tests, it is possible to distinguish five phases in the density evolution. Figure 17a shows the typical evolution of density in each zone during a charge, this image is a general representation of all the tests done and the five phases are indicated. A real time measurement can be observed in Figure 17b; the initial state of the battery corresponds to the steady state. It can be notice the correlation with the graphic of Figure 17a.

The battery SoC is specially difficult to determine when the battery is under a charging process. This is due to the fact that charging process makes the density to not change equally at all points of the battery. During the discharging process the density varies in a linear way with the SoC in all zones in the battery, therefore if we graphically represent the variation of the density in the four zones specified during the discharging process, we would obtain four descending lines. When observing the evolution of the density during the charging process (Figure 17a), five phases may be distinguished for both fast charge and medium charge:

- Phase 1: This is the longest phase. Its main characteristic is that the density varies linearly in the four zones of the battery, although with different slopes. The greatest slope is that of the middle and upper zones, whereas the slope in the curve of the top zone is practically zero.
- Phase 2: In this phase a small bubbling appears which makes the density decrease drastically in the upper zone, whereas in the top zone, the slope begins to increase. In the middle zone there is a positive slope, but less than in phase 1. The slope of the density in the bottom zone does not vary.
- Phase 3: In this phase the density of the middle zones begins to decrease, whereas the density in the upper zone begins to stabilize. The slope of the density in the top zone continues to increase and the bottom zone does not change.
- Phase 4: The density of the middle zone decreases dramatically and that of the top zone increases considerably. The density of the upper zone increases again, after the sharp decrease in the two previous phases.
- Phase 5: This phase is observed in a few tests only. During this phase, the density in the bottom zone decreases, and it increases in the other zones. In Figure 17b, this phase can be clearly seen in the real time measurement.

From the samples taken by the multipoint sensor, and taking into account other parameters, the SoC of the battery may be estimated. The multi-point sensor monitors, in real time, the battery density at different heights and there is not necessary to wait for the density of the liquid inside the battery to become homogeneous. That is a significant improvement in the multi-point sensor in contrast with sensors which only measure the density in the upper-top part of the battery.

### 4.2. Electrolyte low-level detection

During the battery operation, there is a loss of water as a result of the evaporation and of the electrolysis in hydrogen and oxygen. It is very important to maintain a suitable level of electrolyte as, apart from acting as a conductor of the electricity, it provides a medium for the heat transfer of the plates. If the level of electrolyte is not enough to cover the plates completely, there will be areas of the plate without electrochemical activity and the heat will concentrate in other areas of the plates. It is therefore necessary to carry out preventive maintenance and periodic checks of the electrolyte level of the batteries, adding water when necessary. In the case of large groups of batteries, the inspection becomes difficult. Therefore, the availability of real-time information about this parameter is very useful.

**Figure 18.**
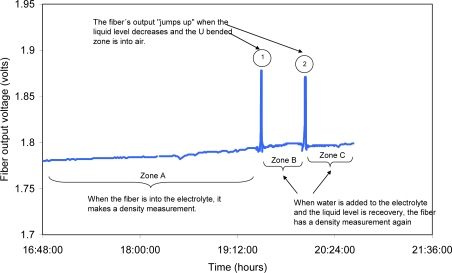
Response of the level fiber as the level of electrolyte diminishes.

Using the top fiber in the multi-point sensor, the level of electrolyte can be monitored in real time. If the fiber is covered by the liquid, it takes measurements of the density, whereas if the level of the electrolyte decreases and the fiber is exposed to the air in the bent zone, there is a noticeable change in the output voltage of the receiver. Figure 18 shows the response of the level fiber during the charging test.

Under normal conditions the fiber takes a measurement of density. In point 1 the output of the fiber jumps because the liquid withdraws from the container of the battery and the fiber remains in the air, the withdrawn electrolyte returns immediately to the container of the battery and the fiber is once again covered and carries out a measurement of density. In point 2, the liquid is taken away and the immediate and noticeable response of the level fiber can be verified. This variation in the output voltage may be used as an alarm and when it occurs, it will mean a low level of electrolyte.

### 4.3. Integrated measurement system

**Figure 19.**
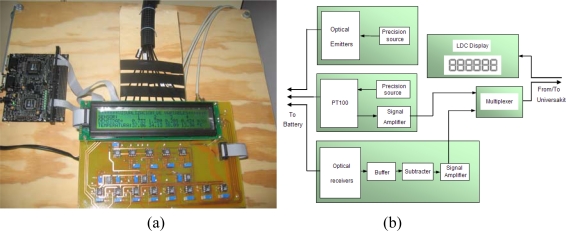
System for conditioning and visualization of the variables of the optoelectronic sensor. (a) Photograph. (b) Block diagram.

An integrated measurement system has been developed. Figure 19a shows the developed system. Figure 19 b shows the circuit block diagram.

This system includes the conditioning and visualization of the variables coming from the multi-point optoelectronic sensors as well as from the temperature sensors. It consists of an electronic circuit including an LCD screen and a Universakit model UVK 0205 development system. The electronic circuit takes the signals from the sensors, adapt their levels and send them to the UVK0205 card. The plate converts these values into data and sends them to the electronic circuit so that they can be visualized on the LCD screen. If a computer with a serial port is available, this data may also be sent to a computer in order to be shown or stored for later analysis.

## 5. Accelerated Testing for Plastic Optical Fibers

In our application, the fibers have to be placed inside the battery cell so it is necessary to know the estimated lifetime. In [11] and [25] the chemical resistance information agrees in which the plastic optical fiber attenuation remains constant when the POF is dipped into sulphuric acid dissolution during 1,000 h and 2,000 h at 50 °C and 60 °C. Taking into account that the battery live can be between 5 and 20 years, depending on the application and the type of battery, it is necessary to design a fiber optic sensor with at least a similar operating life. For these reasons, a 7,000 hours accelerated testing at 70 °C was done in order to estimate the lifetime of the fibers in an electrolytic corrosion environment [30].

Four standard polymer optical fibers (1 mm, 2.2 mm with the jacket) were introduced in a test tube. The fibers were dipped in sulphuric acid with a concentration of 35%. This concentration is similar to the maximum electrolyte concentration (full charge). The test tube is kept in a climatic chamber at 70 °C. Although the test tube was closed, there was a small evaporation of water. This loss of water was compensated refilling the test tube with distilled water. This way the initial level is restored avoiding a change in acid concentration that would modify the density of the solution.

**Figure 20.**
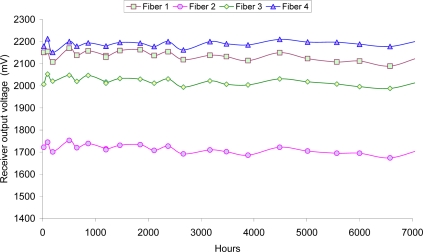
Receivers’ output voltage along the accelerated test for polymer optical fiber dipped in acid sulphuric 35%.

Periodically, transmission measurements were taking in order to detect attenuation changes due to a possible degradation of the material. The fibers were connected to an emitter and a receiver. The receiver output voltage was measured under two conditions: with the LED working and with the LED disabled. In Figure 20 it can be seen the trend of the receivers output voltage for the four fibers. The results indicate that they remain almost constant in the time.

In order to analyze the results, the Arrhenius Model is applied [31]. Considering the conservative results, we had simulated a useful lifetime of 25.6 years at 20 °C, or 12.8 years at 30 °C without increment of transmission losses, that is, without degradation. This lifetime estimation is considered good enough for the sensor application.

## 6. Conclusions

In this article a sensor for the measurement of the electrolyte density at different heights inside lead-acid batteries was described. The experiments carried out with the sensor showed a good behavior, for slow and fast charging as well as slow and fast discharging. Some problems have been found, related to the effect of temperature on the devices. However, these problems have been solved using both, hardware and software systems. This way, the sensor can be placed in many different environments. The difference in densities between the water and the sulphuric acid of the electrolyte show the importance of taking measurements at different heights inside the battery. This way, the multi-point sensor allows us to assets the electrolyte density with higher accuracy.

In addition, the multi-point sensor includes the ability of detecting a low electrolyte level by means of the top measurement point. An electronic system for conditioning, visualization and sensor information storage has been developed, both for density and for temperature of the electrolyte, which constitutes an integrated management system for batteries, including service.

The system allows the connection to a PC where the fundamental data related to the use of the battery can be stored, creating an information record. This information is very useful for the management of banks of batteries. For example, the selection of a charge strategy (slow, fast) can be applied for a particular battery using its own record.

The massive use of this type of sensor in batteries would imply its integration in the battery itself, during the manufacturing process. This type of development may achieve, at a reasonable cost, the transformation of a conventional lead-acid battery into an intelligent battery, which would aid its management throughout its life span.
